# Optimizing immersive learning in crisis management: cognitive and psychological mechanisms of VR training

**DOI:** 10.3389/fpsyg.2025.1695101

**Published:** 2026-01-19

**Authors:** Xiaoquan Tang, Norlaila binti Abdullah Chik, Lei Tang

**Affiliations:** 1 Shanghai University of Medicine and Health Sciences, Shanghai, China; 2 Ghazali Shafie Graduate School of Government, University Utara Malaysia, Sintok, Malaysia; 3 School of Public Health, Shanghai University of Medicine and Health Sciences, Shanghai, China

**Keywords:** cognitive load, decision-making skills, educational psychology, immersive learning, presence theory, training duration, virtual reality training

## Abstract

**Background:**

Virtual Reality (VR) training holds significant promise for crisis education, yet the cognitive and psychological mechanisms through which it enhances higher-order decision-making skills remain inadequately explained. Grounded in Presence Theory and Cognitive Load Theory, this study investigates how VR training improves decision-making competence, examining the mediating role of immersion and the moderating effect of training duration.

**Methods:**

A cross-sectional survey was conducted with 352 emergency response professionals from China, selected via stratified random sampling. Data were analyzed using a combination of Structural Equation Modeling (SEM) and Hayes’ PROCESS macro (Model 59) to test a moderated mediation model.

**Results:**

The model demonstrated substantial predictive accuracy, explaining 35.1% of the variance in immersion and 23.8% in decision-making competence. VR training positively predicted decision-making (*β* = 0.29, *p* < 0.001), with immersion serving as a significant partial mediator [indirect effect *β* = 0.094, 95% CI (0.036, 0.152)]. Training duration marginally moderated the immersion–decision-making link (*β* = −0.14, *p* = 0.054), with simple slopes and Johnson-Neyman analyses confirming that the benefits of immersion are strongest at shorter durations and diminish as training extends.

**Conclusion:**

This study empirically validates immersion as a key psychological mechanism in VR-based learning and identifies training duration as a temporal boundary condition shaping its effectiveness. Theoretically, it extends Presence Theory by linking immersive experience to higher-order cognitive performance; practically, it informs the design of VR-based training policies by emphasizing the coordination of cognitive load, temporal factors, and human–VR interaction design to optimize learning outcomes.

## 1 Introduction

In recent years, immersive learning technologies have gained increasing attention in educational psychology because of their ability to enhance engagement, cognitive processing, and motivation (Brunetti et al., 2024). Among these technologies, Virtual Reality (VR) has emerged as a particularly powerful tool, offering interactive, authentic, and safe environments for learning complex tasks (Tene et al., 2024). VR is defined as a computer-generated, three-dimensional simulation that allows users to interact with and experience an artificial environment, creating a strong sense of presence or psychological immersion (Makransky and Mayer, 2022; Triberti et al., 2025). Presence is a subjective feeling of “being there” in a virtual environment (Makransky and Petersen, 2021; Triberti et al., 2025), and is central to understanding how VR influences learning outcomes (Luna et al., 2023). Research shows that presence and immersion enhance motivation, attention regulation, and cognitive engagement, leading to improved performance and knowledge transfer (Kubr et al., 2024; Li et al., 2023).

Crisis education is one of the most promising domains for applying VR-based immersive learning (Evain et al., 2025). It focuses on developing decision-making, adaptability, and emotional regulation under high-pressure and uncertain conditions (Alshowair et al., 2024; Kubr et al., 2024). Traditional tabletop exercises or classroom lectures, though cost-effective, often fail to reproduce the urgency and complexity of real crises, limiting trainees’ ability to transfer knowledge to real-world action (Muñoz et al., 2024). By contrast, VR provides safe, repeatable, and emotionally realistic crisis simulations that strengthen situational awareness and cognitive readiness without physical risk (Díaz-Barrancas et al., 2025; Mustaqim, 2025). However, while prior studies have demonstrated VR’s benefits for technical and procedural learning, the mechanisms through which VR enhances higher-order cognitive skills like decision-making remain less understood.

From an educational psychology standpoint, VR learning operates through two interconnected mechanisms: *cognitive mechanisms* (information processing, attention focus, and working memory efficiency) and *psychological mechanisms* (emotional engagement, intrinsic motivation, and presence) (Abdelhay, 2024; Makransky and Mayer, 2022). Presence Theory (Steuer, 1993) posits that immersion heightens learners’ sense of realism, emotional investment, and task involvement. When learners experience a strong sense of presence, they allocate greater cognitive resources to the learning task, resulting in deeper understanding and more effective decision-making (Jung, 2022; Makransky and Lilleholt, 2018). In crisis contexts, immersion fosters situational awareness and emotional regulation—key components of effective crisis management (Triberti et al., 2025). Building on this theoretical foundation, prior studies have confirmed that immersion functions as a mediating mechanism linking VR training design to learning outcomes. For example, Abdelhay (2024) demonstrated that immersion and engagement jointly mediate the relationship between VR-based instructional design and training effectiveness, emphasizing how immersive realism enhances hands-on practice and cognitive skill transfer. Together, these findings substantiate immersion’s pivotal mediating role between virtual training environments and cognitive performance. Therefore, the present study hypothesizes:

*H1:* VR training positively predicts decision-making skills.

*H2:* Immersion mediates the relationship between VR training and decision-making skills.

Another critical, yet underexplored, factor is training duration. According to Cognitive Load Theory, learning efficiency depends on managing intrinsic and extraneous cognitive load.

Recent studies emphasize that training duration is a key temporal factor influencing the effectiveness of immersive VR learning. Moderate exposure tends to maximize engagement and presence, while prolonged sessions can cause fatigue and reduce immersion (Yu et al., 2024). Similarly, the positive effects of VR training on decision-making competence may plateau or even decline once the optimal duration is exceeded (Rao et al., 2024). Furthermore, immersion appears to enhance decision performance most effectively within short-to-moderate sessions, but this relationship weakens during extended exposure due to cognitive overload (Hoek et al., 2025).

**Figure 1:**
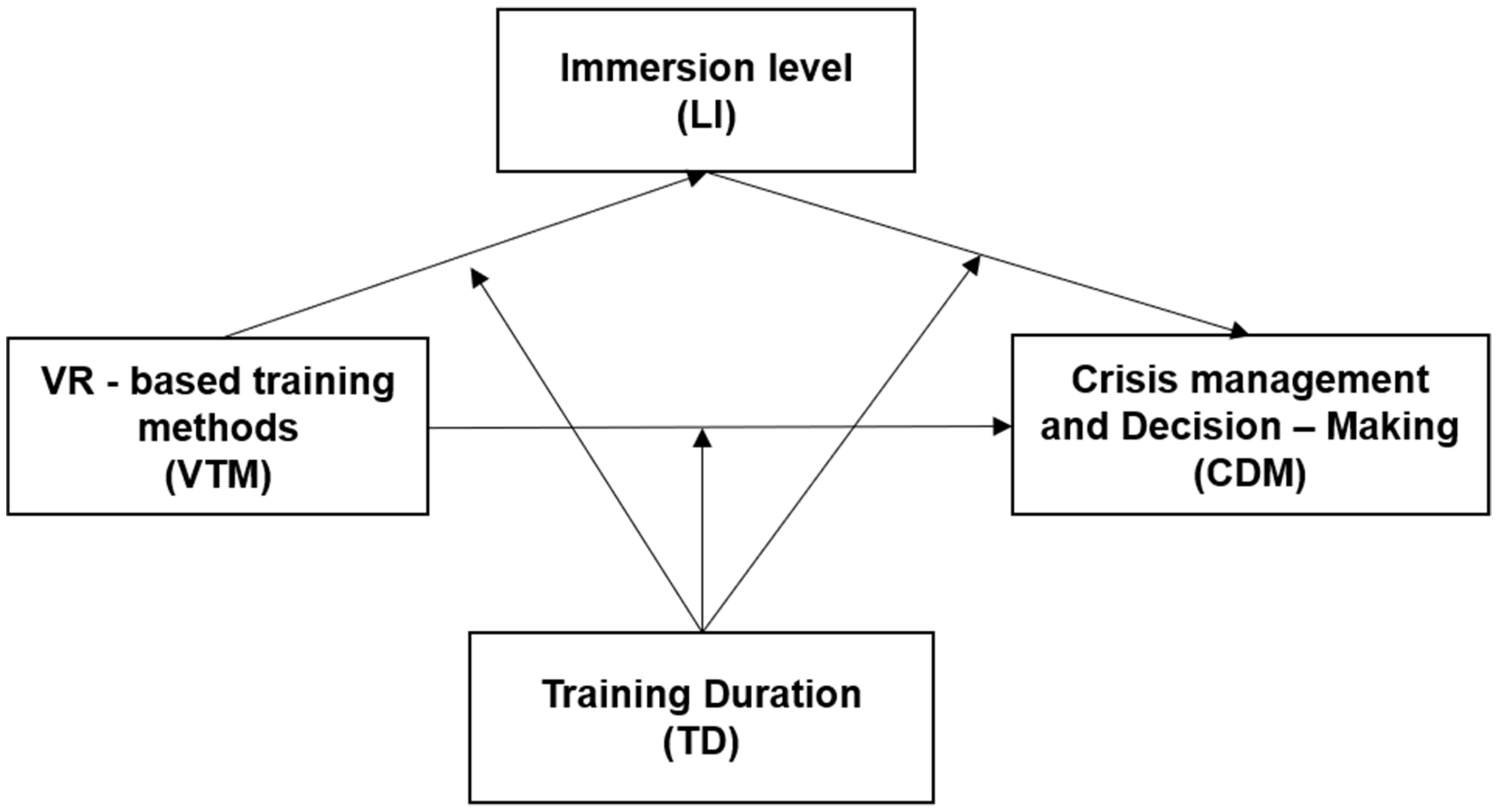
Hypothesized model.

Thus, this study explores the moderating effect of training duration on VR learning outcomes (Figure 1):

*H3:* Training duration moderates the association among VR training, immersion, and decision-making.

By integrating Presence Theory and Cognitive Load Theory, this study seeks to uncover how immersive VR environments enhance decision-making in crisis education. It contributes to the literature by (1) extending Presence Theory to explain higher-order learning outcomes, (2) identifying training duration as a temporal boundary condition affecting immersion, and (3) offering evidence-based implications for designing more effective immersive learning experiences in crisis management training.

## 2 Materials and methods

### 2.1 Participants and procedure

This study employed a cross-sectional design to explore the associations among VR training, immersion, and decision-making competence. This design remains widely used in educational psychology and technology-enhanced learning to examine complex perceptual and behavioral constructs without experimental manipulation (Chevalier et al., 2025; García-Pazo et al., 2023).

Participants were selected using a stratified random sampling approach to ensure representation across regions, professional roles, and training backgrounds. Stratified sampling enhances external validity by controlling subgroup heterogeneity, a strategy increasingly employed in VR and simulation-based education research (Barsac et al., 2024; Khalid et al., 2024). This method remains a best practice for collecting survey data from professionals distributed across multiple institutions or demographic strata, improving precision and minimizing sampling bias.

Data collection was conducted between June and September 2024. The online survey was distributed to training participants via the internal communication platforms of the collaborating training institutions to ensure controlled and targeted access.

The sample size was determined using the method proposed by Krejcie and Morgan (2014), which recommends a sample size of 375 for a population of 15,000 to ensure reliable representativeness at a 95% confidence level with a 5% margin of error, this approach remains widely accepted in recent empirical research for its robustness and comparability across contexts (Nanjundeswaraswamy and Divakar, 2021). However, as noted by Bujang (2021), an additional 40% of questionnaires should be distributed to account for potential non-response and achieve the desired response rate (Bujang, 2021). Based on this recommendation, the final sample size for all selected agencies was adjusted accordingly.

A total of 550 questionnaires were distributed, and 390 responses were received, yielding a response rate of approximately 70.9%. Upon careful review of the collected data, several issues were identified, including missing responses, patterned or zigzag response behavior, and uniform answers across multiple items. After systematically screening and excluding problematic responses, a final dataset comprising 352 valid responses was retained for subsequent analysis.

### 2.2 Instrument development

In consideration of the large-scale survey context, the wording of certain items was refined for clarity while maintaining conceptual consistency, and responses were collected using the same five-point Likert format (1 = strongly disagree to 5 = strongly agree), consistent with current psychometric recommendations emphasizing balance between response precision and cognitive simplicity (Curelaru and Curelaru, 2024; Kusmaryono and Wijayanti, 2022).

The independent variable, virtual reality (VR) training method, was measured with reference to the instrument developed by Oliveira et al. (2023) for assessing the effectiveness of VR-based training. This scale captures trainees’ perceived levels of stress, confidence, and situational awareness experienced in a VR training environment. The mediating variable, *level of immersion (LI)*, was evaluated through participant-based perceived measures. In current VR research, perceived immersion is most commonly assessed using validated self-report questionnaires (Kluge et al., 2023). Accordingly, immersion in this study was measured using a modified version of the *Immersive Technology Experience Measure (ITEM)* proposed by Jacobs, which evaluates user attention, interactivity, and sense of engagement (Jacobs et al., 2023). The dependent variable was defined as decision-making competence, a core component of non-technical skills. Measurement items were developed with reference to the elements and categories identified in recent VR-based competence frameworks, focusing on improvement in balancing risks, selecting appropriate options, gathering relevant information, and making effective decisions (Chan et al., 2024). In particular, immersive learning environments provide a safe and repeatable context to practice judgmental skills, facilitating transfer to real-world crisis and clinical settings (Harris et al., 2023). Additionally, a five-item scale was used to examine the perceived impact of training duration on these competencies.

### 2.3 Ethical considerations

Participation was voluntary, and respondents were assured of anonymity and confidentiality. No personally identifiable information was collected. All participants provided written informed consent prior to participation.

### 2.4 Statistical analysis

To assess the reliability and validity of this study, participants’ demographic characteristics were first examined. Reliability analysis was conducted using SPSS 26, and average variance extracted (AVE) values were calculated to assess convergent validity. Confirmatory factor analysis (CFA) was performed using AMOS 26. Following the procedure outlined by Hayes, PROCESS analysis was employed to test the hypothesized relationships (Hair, 2014). Specifically, Model 59 of the PROCESS macro was used to explore a moderated mediation model, examining the direct effects (H1: VTM–H2: CDM), the mediating effect (H1: VTM–H2: LI–H3: CDM), and the moderating role of training duration on the relationship between VR training and the acquisition of decision-making skills.

### 2.5 Analytical framework and model specification

The hypothesized relationships were tested within a Structural Equation Modeling framework. The model comprises a measurement component, which links the latent variables to their observed indicators, and a structural component, which specifies the relationships among the latent variables.

The structural model is formally represented by the following equation system:

*η* = Bη + *Γξ* + *ζ* Where:

- η is the vector of endogenous latent variables (Level of Immersion - LI, and Competence in Decision-Making - CDM).
- ξ is the vector of exogenous latent variables (Virtual Training Method - VTM, and Training Duration - TD).
- B is the matrix of path coefficients between endogenous variables.
- Γ is the matrix of path coefficients from exogenous to endogenous variables.
- ζ is the vector of disturbance terms.

The moderated mediation relationships, consistent with Hayes’ PROCESS Model 59, are embedded within this framework. The model was estimated using the Maximum Likelihood (ML) method in AMOS 26, which provides efficient and consistent parameter estimates under multivariate normality assumptions.

## 3 Results

### 3.1 Demographic profile

A total of 352 valid questionnaires were collected from emergency response professionals across China’s five major geographic regions.

Gender distribution was predominantly male (M = 1.21, SD = 0.41), with men accounting for 78.7% (*n* = 277) and women 21.3% (*n* = 75).

Age distribution was relatively balanced (M = 2.95, SD = 1.42; valid *N* = 351), with the majority of respondents aged between 25 and 54 years, reflecting the core professional age range. Specifically, 21.4% were aged 25–34, 20.2% were 35–44, and 19.7% were 45–54. Respondents aged 55 and above represented 38.8% of the sample (19.4% each for both the 55–64 and 65 + age groups).

Occupational roles were primarily composed of logistics coordinators (38.1%, *n* = 134) and emergency response officers (35.8%, *n* = 126), followed by crisis management directors (13.9%, *n* = 49) and medical personnel (12.2%, *n* = 43) (M = 2.04, SD = 1.02).

Work experience showed a polarized distribution (M = 3.51, SD = 1.39; valid *N* = 351). Respondents with over 10 years of experience constituted 36.2% (*n* = 127), while those with 4–7 years collectively accounted for 38.5% (*n* = 135†). Only 9.4% (*n* = 33) had less than one year of experience.

Regional representation was broad (M = 3.01, SD = 1.36), with higher proportions from western (23.3%, *n* = 82), eastern (20.7%, *n* = 73), and southern (20.7%, *n* = 73) regions, while northern (17.9%, *n* = 63) and central (17.3%, *n* = 61) regions were comparably represented.

Data quality was high, with only two missing responses (one each for age and work experience), resulting in an overall missing rate of just 0.3%. The variance across variables ranged from 0.168 (gender) to 2.029 (age), indicating acceptable intergroup heterogeneity (Table 1).

**Table 1:** Participant demographics and professional characteristics (*N* = 352).

Gender	*n*	%	Job title	*n*	%	Age (years)	*n*	%	Experience (years)	*n*	%
Male	277	78.7	Logistics coordinator	134	38.1	25–34	75	21.6	> 10	127	36.1
Female	75	21.3	Emergency response officer	126	35.8	35–44	71	20.2	4–7	69	19.6
			Crisis management director	49	13.9	45–54	69	19.7	8–10	56	15.9
			Paramedic	43	12.2	55–64	68	19.6	1–3	66	18.8
						≥ 65	68	19.4	< 1	33	9.4

### 3.2 Common method variance

Exploratory factor analysis revealed that the first factor, as the dominant dimension, accounted for 34.64% of the total variance (eigenvalue = 4.503), which is below the 50% threshold. This suggests that the risk of common method bias remains within an acceptable range (Polas, 2025).

### 3.3 Confirmatory factor analysis

The psychometric properties of the measures were examined through confirmatory factor analysis (CFA) based on the four-factor model, namely VTM, LI, CDM, and TD, The results showed that these measurement items were acceptable.χ^2^(78) = 2457.306, *p* < 0.001; comparative fit index (CFI) = 0.910; Tucker–Lewis index (TLI) = 0.895; root mean square error of approximation (RMSEA) = 0.095; goodness of fit index (GFI) = 0.921; adjusted goodness of fit index (AGFI) = 0.898; and incremental fit index (IFI) = 0.921.

### 3.4 Reliability analysis

The structural model demonstrated satisfactory reliability. As shown in Table 2, the Cronbach’s *α* coefficients for all latent variables exceeded the recommended threshold of 0.70 proposed by Yan et al. (2024), indicating acceptable internal consistency.

**Table 2:** Construct reliability and confirmatory factor analysis of the measurement model.

Factor	Factor loadings	SE	*t*	*P*	Standardized factor loadings	Cronbach’s alpha
VTM1	1	-	-	-	0.773	0.845
VTM3	1.17	0.073	15.928	0.000	0.884	
VTM2	1.133	0.079	14.431	0.000	0.768	
DT1	1	-	-	-	0.657	0.796
DT2	1.207	0.112	10.797	0.000	0.737	
DT3	1.314	0.118	11.165	0.000	0.800	
DT4	1.19	0.122	9.750	0.000	0.638	
LI1	1	-	-	-	0.729	0.723
LI2	1.168	0.095	12.293	0.000	0.875	
LI3	0.808	0.086	9.372	0.000	0.549	
CDM7	1	-	-	-	0.991	0.909
CDM8	0.885	0.031	28.792	0.000	0.893	
CDM9	0.833	0.041	20.319	0.000	0.770	

The hyphen ‘-’ indicates that this item is a reference item.

### 3.5 Convergent validity and discriminant validity

Convergent validity was assessed to confirm the internal consistency of the measurement model. As summarized in Table 3, all constructs demonstrated satisfactory convergence, with average variance extracted (AVE) values exceeding 0.50 and composite reliability (CR) values ranging from 0.768 to 0.918, surpassing the recommended threshold of 0.70. For the DT construct, although its AVE was marginally below the conventional cutoff (AVE = 0.505), it was retained because the standardized factor loadings were all significant and ranged from 0.65 to 0.80—well above the minimum acceptable level of 0.60—and the CR exceeded 0.80. Such conditions satisfy accepted criteria for convergent validity in confirmatory factor analysis, where factor loadings, AVE, and CR collectively demonstrate adequate construct convergence (Hair et al., 2021; Whittaker and Schumacker, 2022).

**Table 3:** Convergent and discriminant validity of the measurement model.

Constructs	(1)	(2)	(3)	(4)	AVE	CR
(1) VTM	**0.810**	-	-	-	0.656	0.851
(2) DT	0.081	**0.711**	-	-	0.505	0.802
(3) LI	0.595	0.055	**0.730**	-	0.532	0.768
(4) CDM	0.461	0.083	0.396	**0.889**	0.791	0.918

1. Bold diagonal values = square roots of AVE (AVE). 2. Off-diagonal values = Pearson correlations between constructs. 3. For discriminant validity, AVE for each construct should exceed all correlations with other constructs.

Discriminant validity was also supported, as the square roots of AVE for all constructs (diagonal elements in Table 3) exceeded the corresponding inter-construct correlations, indicating sufficient construct distinctiveness.

#### 3.5.1 Model fit evaluation

The model fit indices indicated that the hypothesized measurement model exhibited an acceptable overall fit to the data. Although the chi-square statistic (χ^2^) was significant—a common outcome with large samples—and the χ^2^/df ratio (31.50) exceeded the ideal range, this index is highly sensitive to sample size and model complexity and thus should not be interpreted in isolation (Kline, 2023). The complementary indices provided stronger evidence of model adequacy: the comparative fit index (CFI = 0.910) and Tucker–Lewis index (TLI = 0.895) approached the recommended 0.90 threshold, while the root mean square error of approximation (RMSEA = 0.095) and standardized root mean square residual (SRMR = 0.083) were within marginally acceptable limits. Taken together, these results suggest that, despite the inflated χ^2^ value, the measurement model demonstrates an adequate overall fit and can be reasonably retained for subsequent hypothesis testing. Following established SEM guidelines, acceptable fit is typically inferred when CFI and TLI exceed 0.90, RMSEA remains below 0.08–0.10, and SRMR is below 0.08 (Hair et al., 2021; Kline, 2023).

### 3.6 Hypotheses testing

Given the acceptable measurement model fit, the hypothesized moderated mediation relationships were further tested using PROCESS Macro Model 59, which allows simultaneous estimation of indirect and conditional effects.

#### 3.6.1 Direct effects (H1)

VR training method (VTM) significantly predicted crisis decision-making skills (CDM) (*β* = 0.29, *p* < 0.001), supporting H1 (Table 4).

**Table 4:** Direct, indirect, and total effects.

Path	Β	SE	*t*	95% CI	*p*
Direct effects					
VTM → CDM	0.290	0.049	5.886	[0.193, 0.386]	***
VTM → LI	0.520	0.038	13.865	[0.447, 0.594]	***
LI → CDM	0.181	0.056	3.218	[0.070, 0.291]	**
Indirect effect (ATM → LI → CDM)					
Bootstrap result	0.094	0.030	3.180	[0.036, 0.152]	**

**p < 0.01, ***p < 0.001.

#### 3.6.2 Mediating effect of immersion (H2)

VTM had a significant positive effect on immersion level (LI) (*β* = 0.52, *p* < 0.001), and LI significantly predicted CDM (*β* = 0.181, *p* < 0.01). The indirect effect of VTM on CDM via LI was significant [*β* = 0.094, 95% CI (0.036, 0.152)], The Variance Accounted For (VAF) was calculated to be 47.0% (VAF = Indirect Effect/Total Effect = 0.277/0.589), indicating a robust partial mediation effect, confirming partial mediation and supporting H2 (Table 4).

#### 3.6.3 Practical significance and effect sizes

To assess the practical significance of the structural paths, we computed Cohen‘s f^2^ effect sizes (Table 5). The results indicated that Virtual Reality Training Method (VTM) had a large effect on the Level of Immersion (LI) (f^2^ = 0.39). However, the direct effects of both VTM and LI on Crisis Decision-Making (CDM) were of small magnitude (f^2^ = 0.12 and f^2^ = 0.05, respectively), suggesting that while these relationships are statistically significant, their unique contribution to explaining variance in decision-making competence, over and above the other variables in the model, is relatively modest.

**Table 5:** Effect size (Cohen’s f^2^) of structural paths.

Path	R^2^ included	R^2^ excluded	f^2^	Effect size interpretation
VTM → LI	0.351	0.100	0.39	Large
LI → CDM	0.238	0.200	0.05	Small
VTM → CDM	0.238	0.150	0.12	Small

#### 3.6.4 Moderating effects of training duration (H3)

The moderating effect of training duration (TD) was partially supported. TD did not significantly influence the relationship between VR training and immersion or between VR training and decision-making, but it marginally moderated the link between immersion and decision-making (*β* = −0.14, *p* = 0.054), indicating a trending moderation effect.

**Figure 2:**
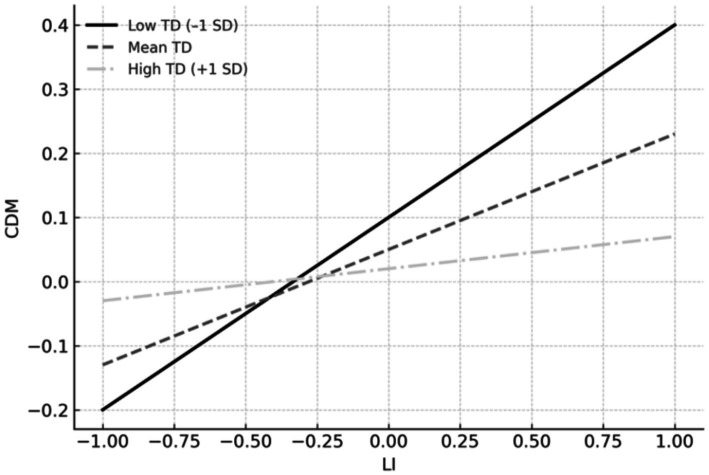
Simple slopes of LI predicting CDM at low (−1 SD), mean, and high (+1 SD) levels of TD.

To probe the moderating role of TD in the association between LI and CDM, a simple slopes analysis was conducted. As depicted in Figure 2, when TD was low (−1 SD), LI significantly and positively predicted CDM (*β* = 0.277, *p* < 0.01), reflecting the strongest effect. At the mean level of TD, the positive association between LI and CDM remained significant but weaker (*β* = 0.209, *p* < 0.05). However, when TD was high (+1 SD), the predictive effect of LI on CDM was substantially attenuated and became non-significant (*β* = 0.073, *p* > 0.05). These results suggest that TD buffers the positive effect of LI on CDM, such that the beneficial influence of LI on decision-making is most pronounced when TD is low.

**Figure 3:**
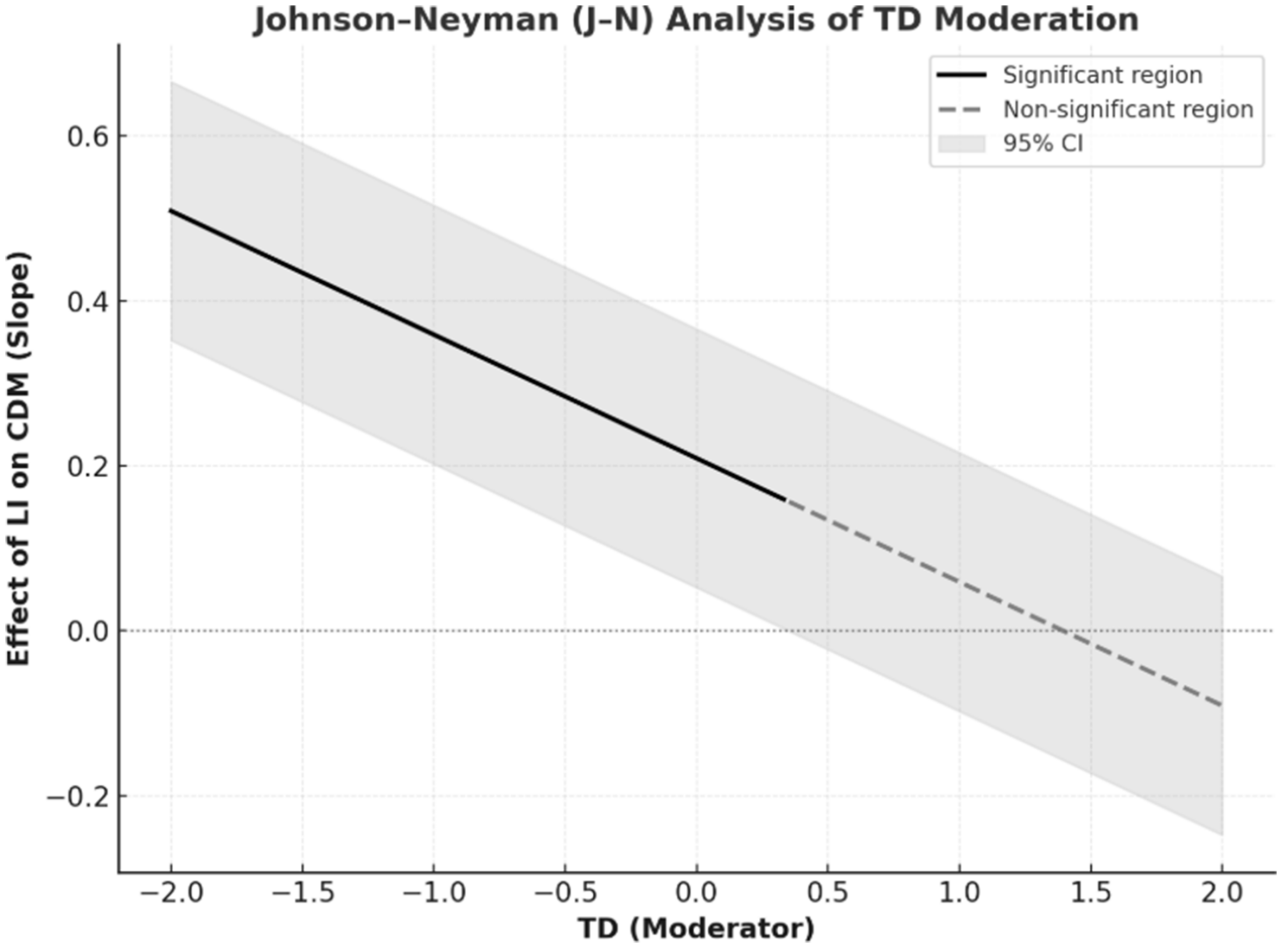
Johnson–Neyman (J–N) analysis of the moderating effect of TD on the relationship between LI and CDM.

To further examine this effect, the Johnson–Neyman (J–N) technique was applied to identify the specific range of training duration values where the moderation effect of TD on the immersion–decision-making relationship became statistically significant. Unlike simple slope analysis, the J–N method provides a more precise understanding of continuous moderation by delineating “regions of significance,” where the predictor’s effect transitions from significant to nonsignificant (Johnson and Fay, 1950). This method has been increasingly adopted in contemporary psychological and educational research using moderated mediation models, offering nuanced insights into how temporal factors shape learning outcomes in immersive environments (Zhu et al., 2022). See Figure 3, The analysis revealed that the positive effect of LI on CDM was statistically significant at lower levels of TD, but gradually weakened as TD increased. At higher levels of TD, the effect of LI on CDM was no longer significant, as the 95% confidence intervals included zero. These findings indicate that the beneficial influence of LI on CDM diminishes with increasing TD, becoming negligible at higher levels of TD.

## 4 Discussion

### 4.1 Summary of main findings

This study investigated how Virtual Reality (VR) training affects decision-making skills in crisis education through cognitive and affective mechanisms, grounded in Presence Theory and Cognitive Load Theory. The results provided mixed but theoretically consistent support for the proposed hypotheses.

#### 4.1.1 VR training positively predicts decision-making skills (H1)

The results confirmed that VR training significantly enhances decision-making skills (*β* = 0.29, *p* < 0.001). This finding aligns with prior research showing that immersive and interactive environments foster experiential learning and critical thinking (Kubr et al., 2024; Li et al., 2023). It also corroborates Jung (2022), who demonstrated that VR-based simulation improves complex decision-making by providing contextual realism and real-time feedback. These results suggest that VR training promotes situational awareness and cognitive adaptability—key components of crisis management competence. Moreover, by extending earlier findings (Evain et al., 2025), this study demonstrates that VR’s benefits persist across different professional groups and crisis scenarios.

#### 4.1.2 Immersion mediates the relationship between VR training and decision-making skills (H2)

The mediating role of immersion [*β*_indirect = 0.094, 95% CI (0.036, 0.152)] supports the theoretical assumption that presence and engagement form psychological pathways translating VR experience into cognitive gains. Mathematically, the significant indirect path (VTM → LI → CDM), supported by bootstrapped confidence intervals that do not include zero, provides robust quantitative evidence for this mediating mechanism. This result is consistent with Triberti et al. (2025) and Makransky and Mayer (2022), who argued that immersion enhances attentional focus and emotional resonance, promoting deeper information processing. However, the current study extends existing knowledge by demonstrating partial mediation, quantified by a Variance Accounted For (VAF) value of 47.0%. This precise metric indicates that nearly half of VR training’s total effect on decision-making is channeled through the psychological pathway of immersion, while the remainder is attributable to direct cognitive and experiential mechanisms. This finding suggests that both direct experiential learning and affective engagement jointly determine decision-making performance. Immersion thus functions not merely as a static emotional experience, but as a dynamic cognitive-affective state whose mediating role is both statistically significant and substantively meaningful.

#### 4.1.3 The moderating effects of training duration (H3a–H3c)

The moderating effects were partially supported. Training duration did not significantly influence the relationship between VR training and immersion (H3a) or between VR training and decision-making competence (H3b). However, a marginal moderating effect was observed on the link between immersion and decision-making (*β* = −0.14, *p* = 0.054), partially supporting H3c. Following the recommendations of *p* values in the 0.05–0.10 range should be interpreted with caution, described as “marginal” or “trending,” and evaluated in the context of theoretical plausibility and effect size rather than as conclusive evidence (McShane et al., 2019). Consistent with this guidance, the present findings suggest a trending moderation effect in which the positive influence of immersion on decision-making competence was stronger at lower levels of training duration and diminished as training duration increased. This trend indicates that extended training may reduce the benefits of immersion due to cognitive fatigue and the decline of attentional engagement over time (Jian and Abu Bakar, 2024; Juliano et al., 2022).

Collectively, these results reinforce that VR training enhances decision-making competence primarily through the mediating role of immersion, with its strength conditionally moderated by training duration. The findings emphasize that the effectiveness of immersive learning depends on optimizing temporal design to balance affective engagement, cognitive load, and sustained presence.

### 4.2 Theoretical implications

#### 4.2.1 Extending presence theory to explain higher-order decision-making performance

This study extends Presence Theory beyond its traditional emphasis on affective and attitudinal outcomes, demonstrating its relevance for higher-order cognitive functions such as analytical reasoning and crisis decision-making. Recent research shows that presence fosters not only emotional engagement but also cognitive engagement and information integration in complex tasks (Kelly et al., 2023). Our results confirm that Virtual Reality Training Methods (VTM) significantly predict learners’ immersion levels, and that immersion, in turn, enhances decision-making skills under crisis conditions. This supports findings that presence serves as a *psychological conduit* transforming sensory and experiential stimuli into cognitive performance (Castronovo et al., 2024; Trevi et al., 2024).

Furthermore, this study provides empirical evidence for the transferability of presence effects across domains—extending from entertainment and gaming contexts to high-stakes environments such as emergency management, medical response, and engineering training. By linking immersive design factors (authenticity, interactivity, multimodal feedback) with measurable decision performance, our findings refine Presence Theory into a more comprehensive model that integrates affective engagement, cognitive investment, and behavioral transfer. This aligns with emerging work positioning presence as a *cognitive-affective bridge* in immersive learning environments (Song et al., 2021).

#### 4.2.2 Introducing training duration as a temporal boundary condition in cognitive load theories

From the perspective of Cognitive Load Theory (CLT), excessively long training durations may lead learners to allocate cognitive resources to non-essential tasks or suffer fatigue-induced declines in processing capacity, thereby weakening immersion’s mediating effect on learning outcomes (Makransky and Mayer, 2022; Sweller, 1988).

Recent empirical evidence substantiates this mechanism: prolonged immersive VR exposure increases cognitive load and decreases long-term retention (Juliano et al., 2022), while high-fidelity visualization, though motivating, can elevate extraneous load and impair performance (Wenk et al., 2023). Similarly, in authentic professional simulations, cognitive load management deteriorates as task interactivity and duration increase (Lämsä et al., 2023).

Studies in adaptive and spatial VR learning contexts further demonstrate that optimal performance emerges under *moderate exposure durations* that balance cognitive challenge and affective engagement (Jian and Abu Bakar, 2024).

Our findings echo these trends: training duration significantly moderates the relationship between immersion and decision-making performance. Immersion exerts stronger positive effects in shorter-duration training contexts but diminishes or disappears with extended exposure. This supports the theoretical refinement of CLT by introducing a temporal boundary condition, suggesting that effective VR-based instruction requires calibrating duration to sustain immersion without exceeding cognitive capacity. These results contribute to the growing body of research optimizing cognitive load and temporal design in immersive learning environments.

#### 4.2.3 Linking immersion dynamics with experiential learning theory

Grounded in experiential learning theory (Kolb, 2014; Makransky and Mayer, 2022), this study extends the theoretical understanding of how immersive virtual environments foster non-technical skills such as crisis decision-making. Our findings demonstrate that immersion exerts a time-sensitive effect: its predictive influence on decision-making is strongest during early exposure, moderates with prolonged practice, and diminishes as skills become consolidated.

This aligns with recent evidence that immersion interacts with training duration and repetition (Jarrah-Nezhad et al., 2024; Rao et al., 2022), suggesting that vivid, engaging experiences are particularly effective for rapid cognitive and reflective learning during the initial learning cycles, while extended practice stabilizes performance regardless of immersion level.

By integrating these insights, our research refines experiential learning theory by introducing the concept of *temporal moderation of immersion effects*—a previously underexplored dimension (Birkheim et al., 2024; Edwards et al., 2023). This theoretical contribution bridges the gap between immersive design and the temporal structure of training, advancing our understanding of how VR-based experiential learning evolves over time.

### 4.3 Practical implications

#### 4.3.1 Policy implications for non-technical skills training

The findings of this study provide empirical support for incorporating VR-based immersive training into national and institutional policies for developing non-technical skills such as crisis communication, decision-making, and teamwork. Evidence from healthcare and public safety sectors shows that immersive simulation policies, when aligned with standardized competency frameworks, can substantially enhance behavioral realism, resilience, and ethical judgment (Gasteiger et al., 2022; Muñoz et al., 2024). For instance, integrating VR into emergency management curricula has been shown to improve psychophysiological regulation and decision-making under stress, supporting policy initiatives that emphasize psychological safety and evidence-based skill certification (Zechner et al., 2023). As highlighted in recent interdisciplinary reviews, strategic policy implementation of VR and AI-based training can foster personalized, ethically informed, and cross-sector learning ecosystems, reducing skill gaps in high-risk domains such as healthcare and public security (Chance, 2025).

#### 4.3.2 Enhancing instructional design through temporal and cognitive coordination

This study further advances instructional design by emphasizing the coordination between immersive experience, training duration, and cognitive load management. Recent empirical studies demonstrate that training outcomes depend not only on immersion intensity but also on the temporal structure of learning—short, highly immersive sessions accelerate skill acquisition, while extended practice consolidates long-term retention (Lin et al., 2025; Pedram et al., 2022). Moreover, adaptive feedback systems and bio-sensor-based monitoring can help educators dynamically adjust cognitive demands to maintain learners’ optimal engagement (Radhakrishnan et al., 2021). This perspective aligns with emerging instructional design frameworks that integrate cognitive load theory and presence-based engagement, emphasizing that immersive learning should not maximize intensity but optimize the balance between mental effort and contextual realism (Dhieb and Durado, 2024).

#### 4.3.3 Promoting interdisciplinary collaboration in human–VR interaction design

Finally, this study calls for systematic interdisciplinary collaboration between educational psychologists, human–computer interaction specialists, and systems engineers to build quantifiable Human–VR Interaction (HVI) models (Joypriyanka et al., 2024). As recent evidence suggests, the next generation of immersive training will rely on data-driven presence metrics—such as real-time cognitive and affective indicators—to personalize learning and enhance decision accuracy (Joypriyanka and Surendran, 2023; Mariselvam et al., 2023). Interdisciplinary frameworks integrating psychophysiology, design science, and learning analytics can enable VR environments to dynamically adapt to users’ psychological states, thereby bridging the gap between theory and practice. Such collaboration is essential to establish standardized, measurable, and ethically grounded guidelines for immersive training system design across educational and professional domains (Tusher et al., 2024).

### 4.4 Limitations and future research

Despite its contributions, this study has several limitations that suggest promising avenues for future inquiry. First, the cross-sectional design restricts causal inference, making it difficult to capture the dynamic evolution of psychological and cognitive mechanisms over time. Future research should employ longitudinal or experimental designs to better establish causal pathways and temporal effects (García-Pazo et al., 2023). Second, the study’s reliance on self-reported data introduces potential common method bias, even though statistical tests were performed to mitigate it (Polas, 2025). Future work should integrate objective, multimodal data streams, including behavioral traces, biometric feedback, and eye-movement analytics, to triangulate findings and minimize subjective distortion. Third, the overall model fit as indicated by the χ^2^/df ratio was poor, likely influenced by the large sample size. Although we relied on a suite of other fit indices to evaluate the model, future research should aim to replicate these findings and consider potential model respecifications to achieve a better fit.

Additionally, the marginally significant moderating effect of training duration warrants further exploration. Future research should examine potential non-linear or threshold patterns, such as an inverted U-shaped relationship, in which moderate training durations maximize immersion and learning transfer, while excessively short or extended exposure may induce cognitive fatigue or diminishing returns.

Finally, moving beyond static VR environments, next-generation studies should employ AI-driven adaptive systems that continuously analyze learners’ physiological and behavioral data to dynamically adjust training complexity, pacing, and content in real time.

## 5 Conclusion

This study provides empirical and theoretical insights into how VR-based immersive training enhances decision-making competence in crisis education. The findings confirm that VR training significantly improves decision-making performance through both direct and indirect pathways, with immersion serving as a key mediating mechanism. Furthermore, the moderating analysis suggests that training duration influences this process: the positive effect of immersion on decision-making is strongest during short-to-moderate training sessions but diminishes with extended exposure, likely due to cognitive overload and fatigue.

Theoretically, the study refines Presence Theory by demonstrating its relevance to higher-order cognitive outcomes and extends Cognitive Load Theory by introducing training duration as a temporal boundary condition. Practically, it provides valuable guidance for policy makers and instructional designers, emphasizing that immersive learning should balance intensity, duration, and cognitive load to sustain engagement and optimize learning transfer. Finally, the study highlights the need for interdisciplinary collaboration among educational psychologists, human–computer interaction specialists, and engineers to advance adaptive, data-driven human–VR interaction models that enable personalized and measurable immersive learning experiences.

Looking forward, this study opens several avenues for future research. First, the marginally significant moderating effect of training duration warrants further investigation into potential non-linear or threshold patterns, such as an inverted U-shaped relationship. Second, longitudinal or experimental designs are needed to track the dynamic evolution of immersion and decision-making skills over time, solidifying causal claims. Third, future work should integrate multimodal data streams (e.g., biometric feedback, eye-tracking) to complement self-reported measures and provide objective validation of cognitive and affective states. Finally, moving beyond static VR environments, next-generation studies should leverage interdisciplinary collaboration to develop AI-driven adaptive systems that can personalize training content and pacing in real-time based on learners’ physiological and behavioral data.

## Data Availability

The datasets presented in this article are not readily available because the dataset is not publicly available due to institutional and confidentiality restrictions but may be obtained from the corresponding author on reasonable request. Requests to access the datasets should be directed to Tangxiaoquan, tang-sh@outlook.com.
